# *Oxalis pes-caprae* L. (Oxalidaceae): From Invasive Concern to Promising Bioresource for Health and Sustainable Applications

**DOI:** 10.3390/plants14040578

**Published:** 2025-02-14

**Authors:** Giuseppe Antonio Malfa, Simone Bianchi, Vivienne Spadaro, Claudia Di Giacomo, Francesco Maria Raimondo, Rosaria Acquaviva

**Affiliations:** 1 Department of Drug and Health Sciences, University of Catania, Viale A. Doria 6, 95125 Catania, Italy; cdigiaco@unict.it (C.D.G.); racquavi@unict.it (R.A.); 2 Research Centre on Nutraceuticals and Health Products (CERNUT), University of Catania, Viale A. Doria 6, 95125 Catania, Italy; 3 PLANTA/Center for Research, Documentation and Training, Via Serraglio Vecchio 28, 90123 Palermo, Italy; raimondo@centroplantapalermo.org; 4 Department of Biological, Chemical and Pharmaceutical Sciences and Technologies, Section of Botany, Anthropology and Zoology, University of Palermo, Via Archirafi 38, 90123 Palermo, Italy; vivienne.spadaro@unipa.it

**Keywords:** sustainability, biodiversity, invasive plant management, natural ingredients, botanicals, polyphenols, oxalic acid

## Abstract

*Oxalis pes-caprae* L., an invasive plant from South Africa, has developed into a severe ecological threat in many Mediterranean and temperate areas by replacing native flora and modifying ecosystems. Although this species has detrimental effects on the ecosystem, it has unrealized potential as a significant bioresource. Current research on the secondary metabolites found in *O. pes-caprae*, such as phenolic acids, oxalates, and flavonoids, is summarized in this review, along with an analysis of their biological and pharmacological properties, which include antibacterial, antidiabetic, and antioxidant activities. *O. pes-caprae* could be converted from a troublesome intruder into a financially and ecologically advantageous bioresource of natural products for pharmaceutical, nutraceutical, cosmetic, and agricultural applications by rethinking the invasive species as a resource for phytochemical extraction. This would offer a novel approach to managing invasive species while promoting the advancement of green technologies and sustainable practices.

## 1. Introduction

Non-native species that aggressively invade new areas, frequently overwhelming native vegetation and upset ecosystems, are known as invasive alien species (IAS) [1]. Native plants are inexorably displaced by these species, reducing biodiversity and putting natural wildlife habitats at risk [1]. In addition, many IAS may reduce agricultural productivity and force farmers to use expensive herbicides, time-consuming removal strategies, and various control techniques, with severe economic and environmental effects [2].

Understanding the potential of invasive species as sustainable economic resources rather than just ecological hazards is critical. Their fast growth, adaptability, and versatility make them valuable raw materials for innovative industries, such as the cosmetic, pharmaceutical, and food industries [3,4,5,6,7].

Currently, the management of IAS occurs through different methods, including mechanical, biological, and chemical processes [8].

In this context, this review aims to assess the potential of *Oxalis pes-caprae* L. (Oxalidaceae) as a sustainable resource. *O. pes-caprae* is a South African perennial, herbaceous, and bulbiferous species that was brought to Europe in the latter part of the 18th century and is today widespread in many warm-climate regions, including the Mediterranean basin, where it is frequently invasive [9]. It is ranked as one of the top ten invasive plants in terms of having the worst effects on the environment and the economy [10].

For this purpose, we provide a brief overview of its botany, ecology, diffusion, nutritional features, and phytochemical profile. We also discuss the main biological activities and recent findings regarding its possible applications in different fields, including the pharmaceutical industry.

## 2. Data Sources

Our investigation in this article is based on multiple reputable sources, including Web of Science, PubMed, Google Scholar, and Scopus. The primary keyword used in this study was the common name *Oxalis pes-caprae* L. and its widespread synonyms, combined with essential terms relevant to the research domain (e.g., morphology, distribution, taxonomy, phytochemicals, biological activity, etc.) to refine and enhance the search results. The bibliography includes 60 references, comprising original research articles, reviews, theses, books, book chapters, and relevant websites, covering publications from 1909 to 2024. Unpublished theses and conference proceedings were excluded from this review. All referenced materials were in either English or Italian, and we accessed them (from 1 May 2024 to 1 December 2024) in whole or through detailed abstracts. Additionally, the chemical structures were cross-referenced using PubChem’s open chemistry database and illustrated with ChemDraw Professional 15.0.

## 3. Botany, Ecology and Distribution

### 3.1. Taxonomy and Morphology

*O. pes-caprae* is a tuberous geophyte belonging to the family Oxalidaceae. Native to South Africa, it has become cosmopolitan in distribution. Various scientific names have been attributed to this species; nowadays, they are considered synonyms. From a taxonomic perspective, *O. pes-caprae* has been assigned several synonyms, including *O. cernua* Thunb., *O. caprina* E.Mey. ex Sond., *O. nutans* Hill, *Acetosella cernua* (Thunb.) Kuntze, and *Bolboxalis cernua* (Thunb.) Small. According to World Flora Online (WFO) [11], seven taxa have been listed under this species, one corresponding to the synonym *Oxalis sericea* Thunb., a taxon now considered a variety. Currently, only two varieties of *O. pes-caprae* are recognized: the nominal variety (*O. pes-caprae* L. var. *pes-caprae*) and *O. pes-caprae* var. *sericea* (Thunb.) T.M.Salter. A form that has become relatively common in the Mediterranean Basin is *O. pes-caprae fo*. *pleniflora* (Lowe) Sunding, which is sometimes treated as a variety under the name *O. pes-caprae* var. *pleniflora* (Lowe) Blanco-Dios [11].

**Figure 1:**
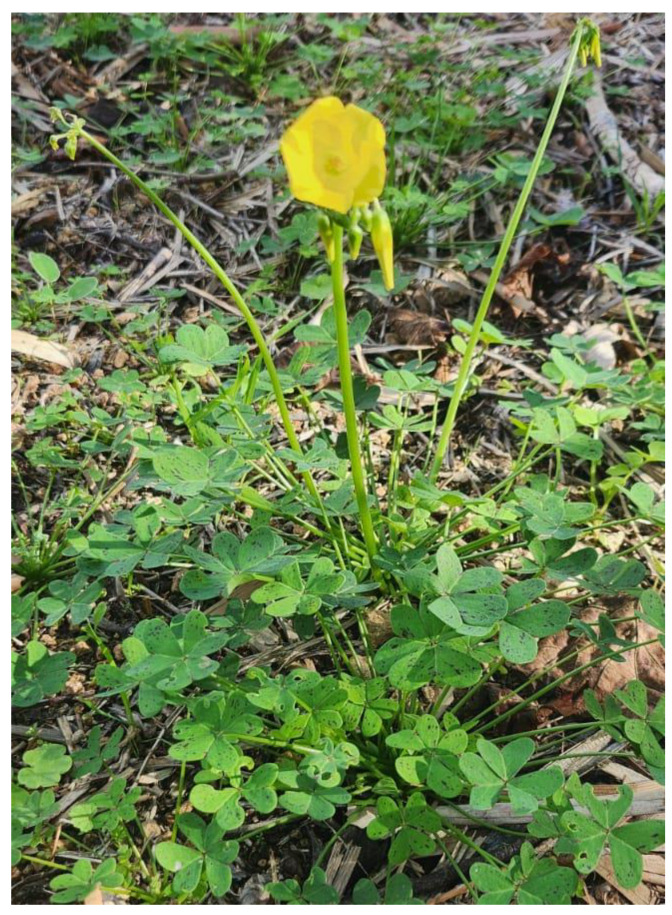
*O. pes-caprae* L. (Oxalidaceae) in flower. Sicily, Italy, December 2024.

This species is a tuberous geophyte with an autumn–winter vegetative cycle. Its trifoliate leaves, borne on long petioles (Figure 1), are supported by underground storage organs including small tubers and bulbils. These organs allow the plant to survive adverse periods and reproduce. In cultivated fields, when unearthed during plowing, they become a sought-after food source for birds and domestic fowl [12]. From a taxonomic perspective, *O. pes-caprae* has been assigned several synonyms, including *O. cernua* Thunb., *O. caprina* E.Mey. ex Sond., *O. nutans* Hill, *Acetosella cernua* (Thunb.) Kuntze, and *Bolboxalis cernua* (Thunb.) Small [13].

### 3.2. Ecology

In South Africa, the plant grows in undisturbed sites and cultivated fields, where it acts as a weed. In its native range, reproduction occurs both sexually through seeds and asexually via tubers and bulbils. In invaded regions, the plants predominantly reproduce vegetatively through these underground organs, although sexual reproduction has recently been observed in the western Mediterranean region [14]. *O. pes-caprae* thrives in light soils but can also tolerate moderately clayey soils. It is a plant of temperate climates, growing from coastal areas to mid-hill elevations [15]. Outside its native range, it vegetates from autumn to late spring during the rainy season. It flowers in mid-winter, and only recently has seed production been observed [16]. The plant grows in cultivated fields when the cultivated species are dormant; thus, its wild populations do not compete with the crops or the native flora associated with these fields [2,17].

**Figure 2:**
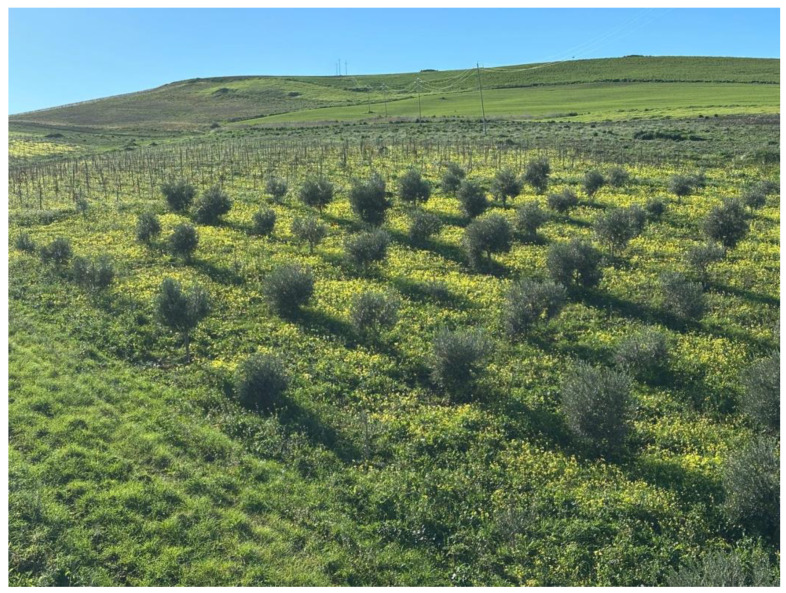
Olive grove and vineyard bordered by a dense growth of yellow *O. pes-caprae* flowers. Sicily, Italy, December 2024.

From a synecological perspective, *O. pes-caprae* forms populations representing the winter facies of the vegetation associated with cultivated fields. These are classified within the vegetation units of the Cl *Stellarietea mediae* Tüxen, Lohmeyer and Preising ex Von Rochow 1951 [18], which includes nitrophilous herbaceous communities characterized by dense coverage and substantial biomass [19]. These communities develop in organic matter and nitrogen soils with a basic pH. This vegetation is ephemeral and nitrophilous, dominated by therophytes and geophytes, and thrives in moist, deep, and shaded soils within thermo- and meso-Mediterranean bioclimatic zones [18]. In Mediterranean countries, *O. pes-caprae* populations are predominantly found in citrus orchards, various fruit tree plantations, intensive olive groves, and all types of row crops (Figure 2). However, from the few pieces of data available in the literature, no significative effects on citrus and olive cultivations have been observed [2,20]. Moreover, cereal and forage crops are rarely or minimally affected, as competition from cultivated species and agricultural practices hinder the plant’s establishment in these areas [17].

### 3.3. Distribution

*O. pes-caprae* is a species native to South Africa (Cape provinces) and Namibia. It was accidentally introduced into various countries across all continents, some of which have seen it become widely naturalized and invasive. According to Plants of the World Online (POWO) [21], the species is recorded in Afghanistan, Albania, Algeria, Argentina Northeast, Argentina Northwest, Arizona, Azores, Balearic Islands, Bermuda, California, Canary Islands, Chile, China, Corsica, Cyprus, Czechoslovakia, East Aegean Islands, Egypt, Eritrea, Ethiopia, Florida, France, Great Britain, Greece, India, Ireland, Italy, Crete, Lebanon, Syria, Libya, Madeira, Mexico, Morocco, New Zealand, Pakistan, Palestine, Peru, Portugal, Sardinia, Sicily, Sinai, Spain, St. Helena, Tasmania, Tunisia, Turkey, Uruguay, and the Western Himalayas. As evidence, within a few centuries, the plant spread from South Africa to all continents, becoming invasive, particularly in regions with a Mediterranean-type climate. These include, most notably, countries in the Mediterranean Basin, USA (California), Chile, and southern Australia. *O. pes-caprae* has thus become a cosmopolitan species, aided by its natural dispersal mechanisms and, more importantly, anthropogenic activities [12]. For instance, the movement of soil from one location to another or the use of earth sourced from areas where the plant grows naturally for transplantation or gardening activities are the primary hidden causes of its spread and accidental introduction to regions beyond its native range.

Southern Africa, its native region, has historically provided many ornamental plants to other continents. These plants were often transported in pots with native soil containing the bulbils and tubers of *O. pes-caprae*, inadvertently allowing their long-distance spread. The Mediterranean region, characterized by a climate similar to South Africa, proved to be an ideal landing point and hub for the proliferation of species within the region and likely other continents [14].

#### Brief History of the Spread of *O. pes-caprae* in the Mediterranean Region

**Figure 3:**
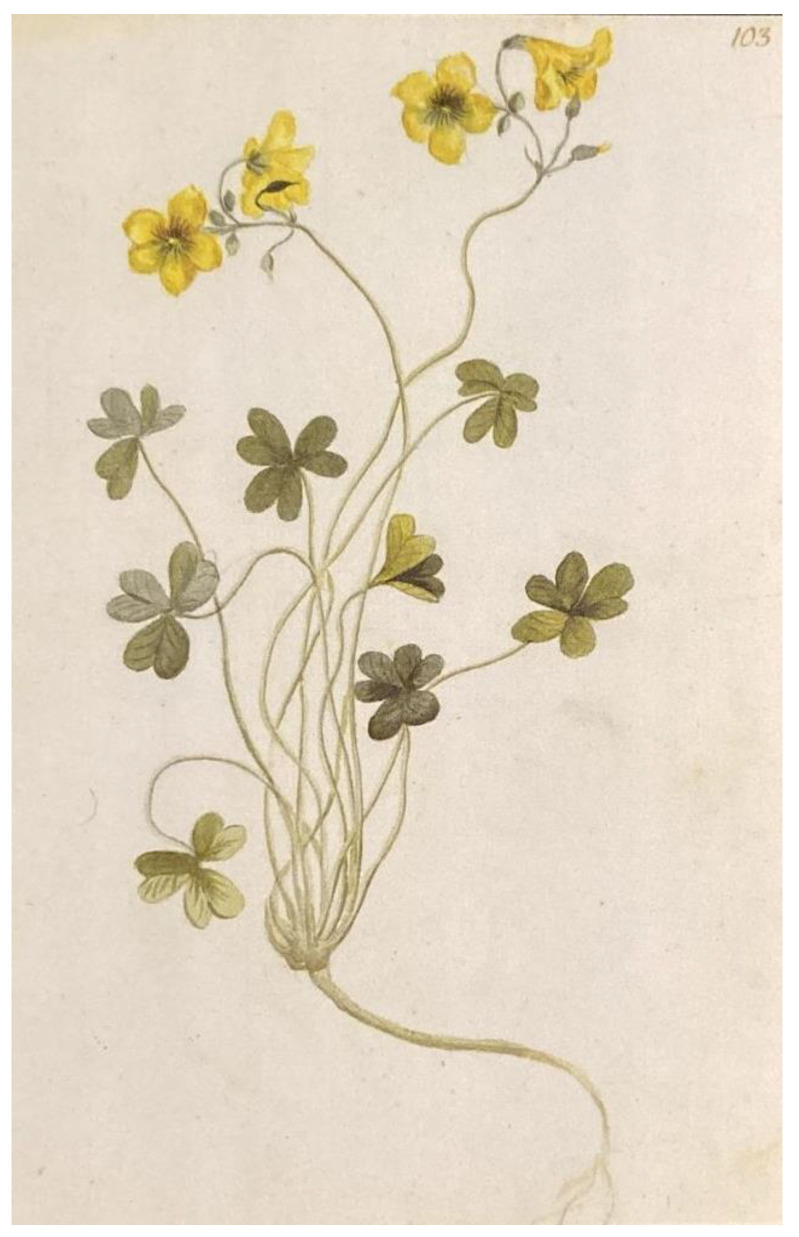
Illustration of *O. caprina* (syn. *O. pes-caprae*), Plate 103 from *Acis Hortus Regius* (1811).

Scholarly attention has focused on the introduction and spread of the species within the Euro-Mediterranean area, with efforts to trace its history. In Italy, for example, its introduction is documented around the late 19th and early 20th centuries [22,23,24]. Various hypotheses exist regarding the introduction and spread of *O. pes-caprae* in Europe, with some studies suggesting a polyphyletic origin for the populations found in Europe and the Mediterranean [14]. Regardless of its initial entry point, one documented location of early establishment was Malta, followed by Italy, where the species was cultivated in some botanical gardens [14]. The interest in introducing *O. pes-caprae* into botanical garden collections still needs to be explored. It is more likely that the species arrived in Europe by transporting other decorative plants from South Africa in pots containing soil rich in bulbils and tubers of *O. pes-caprae* [14]. Contrary to commonly held beliefs, the first documented appearance of the species in Italy predates the late 19th century. It was observed at the beginning of the century. The plant was depicted in the botanical garden of the pharmacist Giuseppe Riggio (1756–1830), located in Acireale, Sicily, in the province of Catania [25]. The body of illustrations depicting the plants hosted in the garden of the aristocratic pharmacist in Acireale was discovered by the renowned Italian publisher Franco Maria Ricci. After acquiring it from an antique dealer in Northern Italy and subsequently publishing it [26], Ricci donated the original manuscript to the library of the Accademia dei Zelanti e dei Dafnici in Acireale, where it is currently preserved. The illustration of *O. pes-caprae*, made by the local artist Emanuele Grasso, corresponds to plate 103 of the first volume and dates back to 1811 (Figure 3).

**Figure 4:**
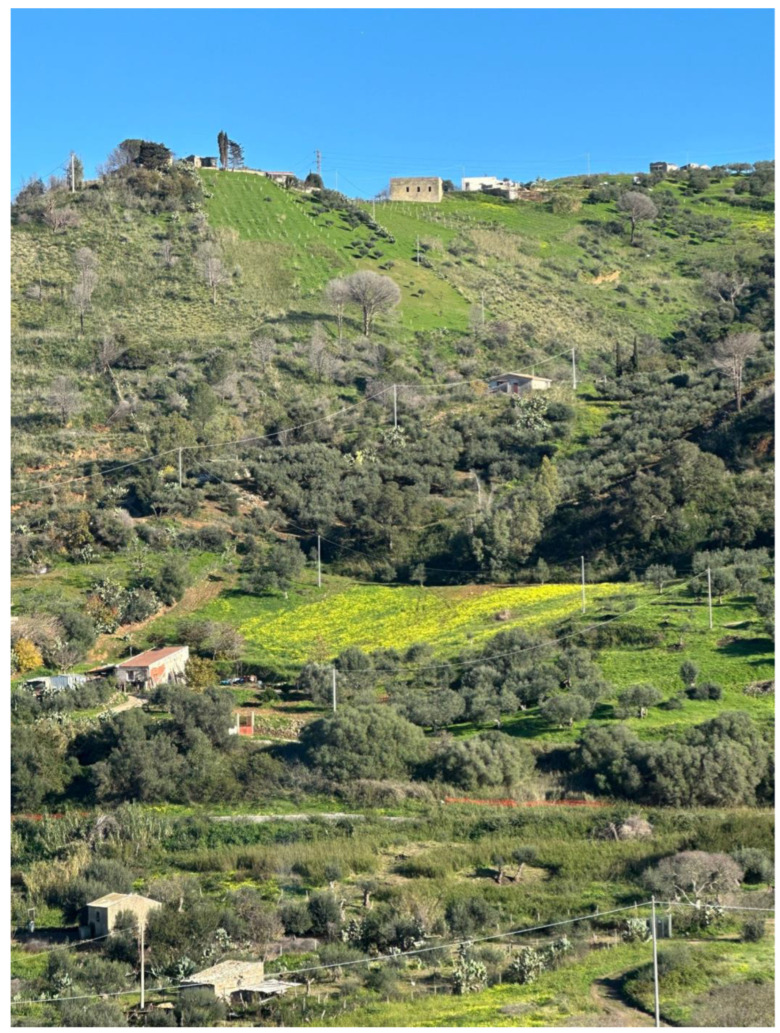
Hilly rural landscape in Sicily, Italy, featuring an extensive bloom of yellow *O. pes-caprae* flowers. December 2024.

The ancient presence in Sicily explains its wide diffusion in the countryside. It is recognizable by admiring the winter agricultural landscapes where the maximum flowering is recorded, perceived as a landscape marked by the geometric yellow of the cultivated fields (Figure 4).

## 4. Nutritive Features

In an era of growing global concerns, such as population increase, biodiversity loss, and climate change, research on edible wild plants is crucial to ensuring the sustainability of food supplies. Additionally, because of their environmental adaptation and nutritional richness, IAS, many of which are still underutilized, have enormous potential as alternative food sources. Understanding their nutritional features can help us diversify food sources and incorporate them into sustainable agricultural systems, turning the expenses associated with controlling these invasive species into financial gains. Reports on the ethnobotanical use of this plant species as a food reveal that humans have consumed the bulbs since the Middle Pleistocene as a good supply of carbohydrates [27]. Even today, this plant is used in the preparation of traditional dishes among the populations of some regions of South Africa [28,29]. The entire plant is edible, but the aerial parts should not be eaten in large quantities due to oxalic acid, whose content is almost insignificant in the dormant bulbs [30]. This compound is the simplest dicarboxylic acid and particularly abundant in these plants. Carl Wilhelm Scheele was the first to isolate this compound from the rhizomes of *Rheum rhabarbarum* L., not from *Oxalis acetosella* L. The confusion may have arisen from a mistranslation of the publication of the Swedish term “*Acetosell-syra*”, which refers to oxalic acid [31]. The consumption of elevated daily doses of oxalate may cause health problems, including reduced bioavailability or sequestration of minerals such as calcium and iron and precipitation of calcium oxalate in the urine and formation of kidney stones [32]. Moreover, elevated oxalic acid plasma levels have been recently correlated with increased risk for cardiovascular diseases [33].

Depending on the application, several methods can be employed to remove oxalic acid from plant matrices [34].

Chemical Precipitation: By adding calcium salts, such as calcium chloride or calcium hydroxide, oxalic acid can be precipitated as insoluble calcium oxalate. This approach is commonly used in food processing and instils confidence in reducing oxalate levels in vegetable products [34,35]. Enzymatic Degradation: Oxalate oxidase enzymes offer a significant advantage in certain applications. They can degrade oxalic acid into less harmful compounds like carbon dioxide and water, thereby minimizing chemical residues and ensuring the safety of the final product [34,36]. Solvent Extraction and Filtration: Organic solvents or acidic extractions, followed by filtration, are methods that find relevance in laboratory settings for analytical purposes [37].

Recently, this wild edible plant species was investigated for its nutritional value in the context of improving nutrition and promoting sustainable agriculture; the approximate composition of the different parts of the plant is reported in Table 1 [38]. The research results highlighted that *O. pes-caprae* is a rich source of minerals such as iron and zinc, Vitamin C, essential and non-essential amino acids, linoleic acid (in the flowers), and α-linolenic acid (in the leaves). The average amount of oxalic acid in the aerial parts was about 100 g kg^−1^ dry weight, with the highest value recorded for stems. This value is not that much distant from that found in other food plants, such as leaves of *Cynara cardunculus* L. var. *altilis* (81 g/kg) [38]. The researchers concluded that thanks to its nutritional characteristics, this species has great potential for development in the agri-food sector [38].

## 5. Phytochemistry

The initial studies on the phytochemical profile of *O. pes-caprae* were conducted by Della Greca and colleagues, focusing on leaves and twigs collected in April in Naples, Italy. The extracts were prepared using ethyl acetate, MeOH, and water as solvents at room temperature for seven days. The authors reported, as phytochemical constituents of their extracts, several compounds belonging to different classes, including flavonoids and their derivatives (tangeretin, nobiletin, 5-demethylnobiletin, 4′-demethylnobiletin), phenolic acids and their derivatives (4′-acetylphenyl 4-hydroxycinnamate, 4′-acetylphenylsinapate, 3′-acetylphenyl 4-methylsinapate, 4′-acetylphenyl 4-O-methylsinapate, 4′-(1-hydroxyethyl)phenylsinapate, 3′-(1-hydroxyethyl)phenyl, 4-methylsinapate, *p*-coumaric acid, dihydrocinnamic acid, *cis-p*-coumaric acid, cinnamic acid, 4-hydroxybenzoic acid, (E)-3-methoxyphenyl 4-hydroxycinnamate, (E)-4-(1-(4-(1-hydroxyethyl)phenoxy)ethyl) phenyl 3,4,5-trimethoxycinnamate), terpenes (loliolide), and others (1,2,3,4-tetrahydro-1-methyl-b-carboline-3-carboxylic acid, benzoic acid, 3-methoxyphenol, 2-methoxyphenol, 4-(1-hydroxyethyl)phenol, 3-(1-hydroxyethyl)phenol, 2-methoxyphenyl 3-phenylpropanoate, 2-hydroxyethyl 3-phenylpropanoate, (E)-4-[4-(2-carboxyethenyl)phenoxy]benzoic acid, 4,4′-[1,1′-oxybis(ethane-1,1-diyl)]diphenol) (Table 2 and Table 3) [39,40,41].

Two extraction procedures were conducted on the aerial parts of *O. pes-caprae*, which were collected in Crete (Greece) in February. In the first one, the plant matrix was kept in ethyl acetate (ratio 1:5) at 40 °C for two hours, then the same matrix was extracted with MeOH (ratio 1:5). In the second one, the plant matrix was infused in boiling water (1:5) for 15 min, then the extract was partitioned with n-butanol. The quantitative analysis showed that the ethyl acetate extract was the richest in phenolic compounds, with 8.46 mg GAEs/g of extract, followed by the MeOH (4.16 46 mg GAEs/g) and the *n*-butanol (3.32 mg GAEs/g) extracts. The authors also performed qualitative LC-DAD-MS (ESI+) and NMR analyses of the extracts, identifying several compounds, such as chlorogenic acid, quinic ferulate, luteolin glucoside, luteolin glucoside isomer, and cernuoside in the ethyl acetate extract, chlorogenic acid, and luteolin C-O-diglucoside in the MeOH extract and chlorogenic acid, luteolin C-glucoside, luteolin C-O-diglucoside, and cernuoside in the *n*-butanol extract (Table 2) [42].

The phytochemical profile of extract from the fresh leaves of *O. pes-caprae* was evaluated using LC-MS-MS analysis, identifying six phenolic compounds: 7,3′-dimethoxyl-2″-O-glycosyl orientin, 7,3′-dimethoxyl-6-desoxyhexose orientin, 6-hexosyl-8-acetylhexosyl luteolin, vitexin, 7,4′-dimethoxyl-2″-glycosyl vitexin, and 2″-O-rhamnosyl vitexin (Table 2). The plant was collected in March in Coimbra (Portugal). The extract was prepared using a hydroalcoholic solution with 50% (*v*/*v*) ethanol (EtOH) at room temperature; the ratio was 1:4. The extract was then dried and resuspended in water. The polyphenolic fraction was extracted with ethyl acetate to remove the oxalic acid from the crude extract [43].

Extracts from different dried parts (flowers, stems, and leaves) of *O. pes-caprae* harvested during the flowering time (March and April) in Bhakkar (Pakistan) were obtained by soaking the plant matrix in methanol (MeOH) or *n*-hexane, at a ratio of 1:10, for one week. The authors of the study reported the presence of different classes of phytochemicals, namely polyphenols, saponins, and alkaloids, as evaluated using qualitative assays in all the plant parts screened. Furthermore, they also carried out quantitative analyses of total phenolic compounds (TPC) and total flavonoid compounds (TFC), finding that their amounts varied considerably among the different parts and solvents used (TPC: 38.55–65.68 mg of gallic acid equivalents (GAE)/g of extract; TFC: 14.83–24.75 mg quercetin equivalents (QE)/g of extract). Specifically, the methanolic flower extracts were found to be the richest in both TPC and TFC [44]. Lahlali and colleagues prepared an aqueous extract of *O. pes-caprae* dried aerial parts, collected in May in Khouribga (Marocco), via digestion under stirring in water at 45 °C for 48 h at a ratio of 1:10. The obtained extract was autoclaved and a quantitative analysis of the TPC was carried out, finding 803 µg GAE/mL of extract, corresponding to 8,03 mg of GAE/g of plant matrix [45].

In a recent study, extracts from *O. pes-caprae* dried leaves and flowers, collected in Tangier (Morocco) and extracted using water or MeOH 80% as solvents at a ratio of 1:10 at room temperature for 24 h, were quantitatively and qualitatively characterized, finding that the methanolic flower extract was the richest in TPC (173.54 mg/g). HPLC-DAD-ESI/MS analysis identified the following phenolic derivatives: luteolin, luteolin-O-dihydrogalloyl-trihexoside, iso-luteolin-O-dihydrogalloyl-trihexosides, kaempferol-di-O-hexosides, kaempferol-O-coumaroyl-hexoside, kaempferol, kaempferol-3-apiosyl-hexoside, isovitexin-O-hexoside, kaempferol-hexoside, kaempferol-di-hexoside, kaempferol-pentose, apigenin-di-C-hexoside, iso-apigenin-di-C-hexosides, and quercetin-O-hexoside (Table 2) [46].

**Figure 5:**
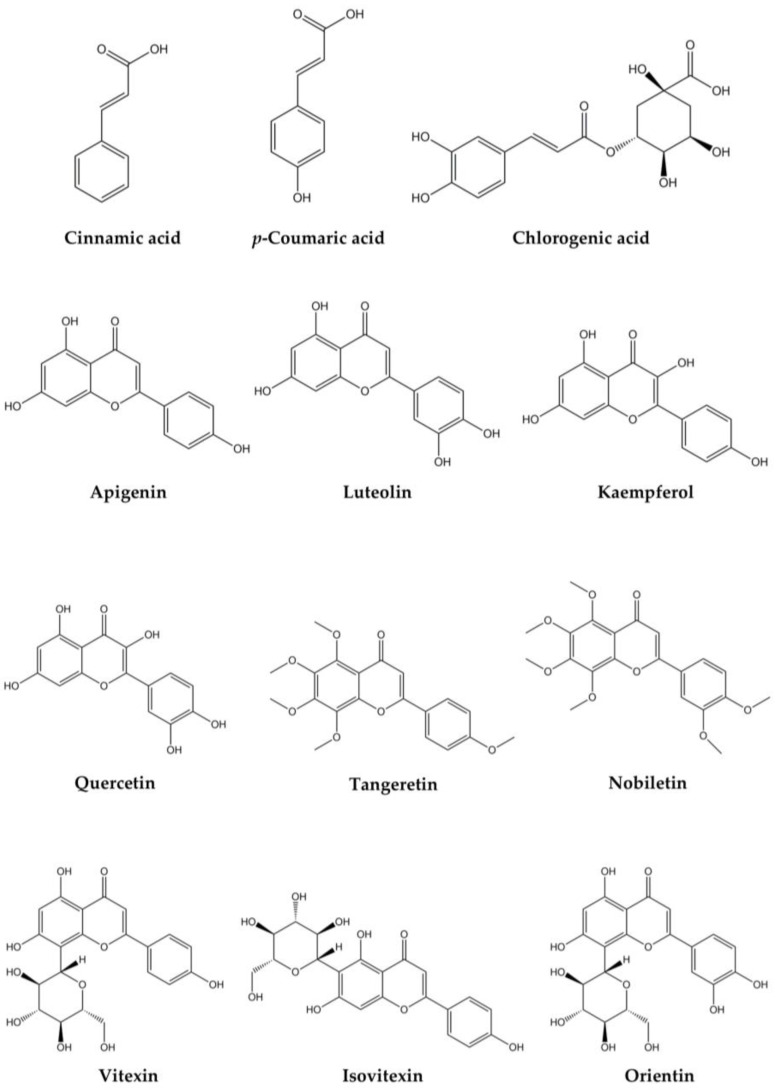
The most representative phytochemicals in *O. pes-caprae*.

The structures of the most representative phytochemicals in *O. pes-caprae* are reported in Figure 5.

**Table 2:** Phytochemicals in *O. pes-caprae* and relative extraction applied.

Extraction	Compounds	Reference
Ethyl acetate, leaves and twigs	4′-acetylphenyl 4-hydroxycinnamate; 4′-acetylphenyl sinapate; 4′-acetylphenyl 4-O-methylsinapate; 3′-acetylphenyl 4-methylsinapate; 4′-(1-hydroxyethyl)phenyl sinapate; 4-methylsinapate	[39]
MeOH, leaves and twigs	(E)-3-methoxyphenyl 4-hydroxycinnamate; 2-methoxyphenyl 3-phenylpropanoate; 2-hydroxyethyl 3-phenylpropanoate; 4,4′-[1,1′-oxybis(ethane-1,1-diyl)]diphenol; dihydrocinnamic acid; 3-methoxyphenol; 2- Methoxyphenol; tangeretin; nobiletin; 5-demethylnobiletin; 4′-demethylnobiletin	[40]
Water, leaves and twigs	(E)-4-[4-(2-carboxyethenyl)phenoxy]benzoic acid; *p*-coumaric acid; *cis-p*-coumaric acid; cinnamic acid; 1,2,3,4-tetrahydro-1-methyl-β-carboline-3-carboxylic acid; 4-hydroxybenzoic acid; 4-(1-hydroxyethyl)phenol; 3-(1-hydroxyethyl)phenol	[40]
Ethyl acetate, leaves and twigs	(E)-4-(1-(4-(1-hydroxyethyl)phenoxy)ethyl) phenyl 3,4,5-trimethoxycinnamate; loliolide	[41]
Ethyl acetate, aerial parts	Chlorogenic acid; Quinic ferulate; Luteolin glucosides; Cernuoside	[42]
MeOH, aerial parts	Chlorogenic acid; Luteolin C-O-diglucoside	[42]
Boiling water/*n*-butanol, aerial parts	Chlorogenic acid; Luteolin C-glucoside; Luteolin C-O-diglucoside; Cernuoside	[42]
Hydroalcoholic (50%), leaves	7,3′-dimethoxyl-2″-O-glycosyl orientin, 7,3′-dimethoxyl-6-desoxyhexose orientin, 6-hexosyl-8-acetylhexosyl luteolin, vitexin, 7,4′-dimethoxyl-2″-glycosyl vitexin, and 2″-O-rhamnosyl vitexin	[43]
MeOH 80%, leaves and flowers	luteolin, luteolin-O-dihydrogalloyl-trihexoside, iso-luteolin-O-dihydrogalloyl-trihexosides, kaempferol-di-O-hexosides, kaempferol-O-coumaroyl-hexoside, kaempferol, kaempferol-3-apiosyl-hexoside, isovitexin-O-hexoside, kaempferol-hexoside, kaempferol-di-hexoside, kaempferol-pentose, apigenin-di-C-hexoside, iso-apigenin-di-C-hexosides, and quercetin-O-hexoside	[46]

In 2024, the volatile compounds in the aerial parts (flowers, leaves, and stems) of *O. pes-caprae* collected in February in Alicante (Spain) were investigated using GC-MS analysis. The results highlighted the presence of 32 compounds belonging to different classes, including aldehydes (3-methyl butanal, nonanal), alcohols (3-hexen-1-ol, phenylethyl alcohol, 1-dodecanol, 1-tetradecanol), esters (4-penten-1-yl acetate, pentyl acetate, isoamyl propionate, 3-hexenyl acetate, hexyl acetate, pentyl butanoate, isoamyl butanoate, 1,1′-bicyclohexyl, ethyl nonanoate, isoamyl benzoate, ethyl dodecanoate, ethyl hexadecanoate), ethers (diisoamyl ether), carboxylic acids (nonanoic acid), terpenes (linalool, α-terpineol, β-caryophyllene, β-farnesene, humulene, nerolidol), and others (1,3-bis(1,1-dimethylethyl)benzene, 4,6-dimethyl dodecane, pentadecane, 2,4-bis(1,1-dimethylethyl)phenol, hexadecane, cyclotetradecane). Specifically, pentyl butanoate, linalool, nonanal, isoamyl butanoate, phenylethyl alcohol, α-terpineol, 1,1′-bicyclohexyl, ethyl nonanoate, β-caryophyllene, isoamyl benzoate, β-farnesene, humulene, and nerolidol were found only in the flowers, 3-hexen-1-ol was found only in the stems, and pentadecane and cyclotetradecane were found only in leaves. Moreover, ethyl dodecanoate was absent only in the stems and 1-tetradecanol only in the flowers (Table 3) [38].

**Table 3:** Volatile compounds of *O. pes-caprae*.

Classification	Name	Reference
Aldehydes	3-methyl butanal	[38]
	nonanal	[38]
Alcohols	3-hexen-1-ol	[38]
	phenylethyl alcohol	[38]
	1-dodecanol	[38]
	1-tetradecanol	[38]
Esters	4-penten-1-yl acetate	[38]
	pentyl acetate	[38]
	isoamyl propionate	[38]
	3-hexenyl acetate	[38]
	hexyl acetate	[38]
	pentyl butanoate	[38]
	isoamyl butanoate	[38]
	1,1′-bicyclohexyl	[38]
	ethyl nonanoate	[38]
	isoamyl benzoate	[38]
	ethyl dodecanoate	[38]
	ethyl hexadecanoate	[38]
Ethers	diisoamyl ether	[38]
Carboxylic acids	nonanoic acid	[38]
Terpenes	linalool	[38]
	α-terpineol	[38]
	β-caryophyllene	[38]
	β-farnesene	[38]
	humulene	[38]
	nerolidol	[38]
	loliolide	[41]
Others	1,3-bis(1,1-dimethylethyl)benzene	[38]
	4,6-dimethyl dodecane	[38]
	2,4-bis(1,1-dimethylethyl)phenol	[38]
	hexadecane	[38]
	pentadecane	[38]
	cyclotetradecane	[38]

## 6. Biological Activities

Several studies have highlighted the different biological activities of *O. pes-caprae*, particularly its antioxidant, antidiabetic, antibacterial, and antifungal activities, as well as its vascular and neuroprotective properties (Table 4).

These benefits are primarily attributed to various polyphenols in the plant’s different aerial parts. Therefore, it is reasonable to consider *O. pes-caprae* as a promising natural source of phytochemicals that could have potential pharmacological applications that could aid in managing this invasive and, by now, naturalized species.

### 6.1. Antioxidant Activity

The scientific literature indicates that extracts from *O. pes-caprae* exhibit strong antioxidant properties. However, these properties are significantly influenced by the extraction technique, the plant part used, and the resulting composition of the extract. Various models have been employed to evaluate antioxidant activity, including in vitro cell-free and in vivo models. The 2,2-diphenyl-1-picrylhydrazyl radical (DPPH•) is a relatively stable radical commonly used for the spectrophotometric evaluation of the antioxidant properties of plant extracts, primarily through the mechanism of hydrogen atom transfer (HAT) [47]. The reported IC_50_ values for *O. pes-caprae* vary widely, ranging from 5.22 to 296 µg/mL [43,44,46,48]. Notably, the lowest value was obtained from acetone leaf extract [48]. Additionally, recent studies have demonstrated that extracts derived from the flowers of *O. pes-caprae* exhibited more potent antioxidant properties, as reflected by lower IC_50_ values, than those obtained from other parts of the plant [44,46].

**Table 4:** Biological activity of extracts of *O. pes-caprae.* GAE: gallic acid equivalent; AAE: ascorbic acid equivalens; N.S.: not specified.

Activity	Extract	Dose	Reference
Antioxidant (DPPH test)	Hydroalcoholic (50%), leaves	IC_50_ 17.9 μg/mL	[43]
	*n*-Hexane, flowers	IC_50_ 24.6 μg/mL	[44]
	Methanol, flowers	IC_50_ 36.4 μg/mL	[44]
	Methanol, leaves	IC_50_ 46.3 μg/mL	[44]
	*n*-Hexane, leaves	IC_50_ 56.2 μg/mL	[44]
	Methanol, stems	IC_50_ 57.1 μg/mL	[44]
	*n*-Hexane, stems	IC_50_ 66.8 μg/mL	[44]
	Aqueous, flowers	IC_50_ 114 μg/mL	[46]
	Methanolic, flowers	IC_50_ 134 μg/mL	[46]
	Methanol, leaves	IC_50_ 214 μg/mL	[46]
	Water, leaves	IC_50_ 296 μg/mL	[46]
	Acetone, leaves	IC_50_ 5.2 μg/mL	[48]
	Methanol, leaves	IC_50_ 17.3 μg/mL	[48]
	Water, leaves	IC_50_ 31.4 μg/mL	[48]
	Ethanol, leaves	IC_50_ 73.2 μg/mL	[48]
	*n*-Hexane, leaves	IC_50_ > 100 μg/mL	[48]
Antioxidant (Fe^3+^ reducing power)	Methanol, stems	34.98 mg GAE/g	[44]
	Methanol, flowers	795.8 mg AAE/g	[46]
	Water, flowers	690.8 mg AAE/g	[46]
	Methanol, leaves	507.8 mg AAE/g	[46]
	Water, leaves	387.4 mg AAE/g	[46]
Antioxidant (Increase in SOD, CAT, GPx, and GR activity in diabetic mice)	Methanol, flower	150–250 mg/kg	[46]
Antioxidant (Increase in DPPH radical scavenging activity in plasma of mice)	Methanol, N.S.	100–200–400 mg/kg	[49]
Antioxidant (Increase of liver GSH in mice)	Methanol, N.S.	100–200–400 mg/kg	[49]
Antidiabetic (Inhibition of α-amylase)	Methanol, flower	Inhibition of 68% at 187.5 µg/mL	[46]
Antidiabetic (Inhibition of α-glucosidase)	Methanol, flower	Inhibition of 40% at 222.5 µg/mL	[46]
Antidiabetic (Inhibition of non-enzymatic glycation)	Methanol, flower	Inhibition ~100% with 1 mg/mL	[46]
Antidiabetic (Increase in carbohydrate metabolic enzyme activity in mice)	Methanol, flower	150–250 mg/kg	[46]
Antibacterial	Ethanol, methanol, acetone, n-hexane, and chloroform, N.S.	1000, 1500, and 2500 ppm	[29]
Antifungal	Ethanol, N.S.	1000 ppm	[29]
Vascular (Reduction of maximum contraction response to noradrenaline)	Hydroalcoholic (50%), leaves	Reduction of 58.4% with 0.66 mg/mL	[43]

The reducing power of *O. pes-caprae* extracts towards iron Fe^3+^, which involves a single electron transfer (SET) mechanism, was also measured in two studies [47]. The results showed that methanolic stem and water leaf extract were the most active among the tested samples [44,46].

The antioxidant properties of *O. pes-caprae* extracts were also evaluated in vivo in mice. Specifically, Kabach et al. used a methanolic flower extract to treat diabetic mice, finding that the treatment was able to counteract hyperglycemia-induced oxidative stress, increasing the activity of the endogenous antioxidant enzymes such as superoxide dismutase (SOD), catalase (CAT), glutathione peroxidase (GPx), and glutathione reductase (GR) in different organs [46]. Belghoul et al. found that the treatment of mice with *O. pes-caprae* methanolic extract increased the overall reducing capability of plasma, measured by the DPPH test, and glutathione (GSH) levels in the liver [49]. Conversely, the treatment decreased the amount of the antioxidant tripeptide in the kidneys of mice and the CAT activity in the liver and kidney was unchanged or even reduced compared to untreated control mice [49]. These results do not contradict the findings from Kabach et al., as they observed an increase in CAT with the treatment compared to diabetic mice, but it was still lower than that in the non-diabetic control group [46].

### 6.2. Antidiabetic Activity

Oxidative stress plays a significant role in the pathophysiology of diabetes. This condition is characterized by increased lipid peroxidation, altered glutathione metabolism, mitochondrial dysfunction, and glycation. Glycation leads to structural and functional changes in proteins, resulting in the formation of high levels of advanced glycation end products (AGEs). These glycated derivatives contribute to the development of microvascular complications, such as retinopathy and nephropathy, as well as macrovascular complications, including cerebrovascular, peripheral vascular, and cardiovascular diseases. Thanks to their phytochemical contents, many medicinal plants have served as effective treatments for diabetes since ancient times [50].

The antidiabetic effects of these plants are linked to their antioxidant properties and ability to inhibit the key enzymes involved in hyperglycemia, specifically α-glucosidase and α-amylase. These two enzymes are essential for breaking down carbohydrates into glucose [28,29,46].

Kabach et al. reported that methanolic extracts of *O. pes-caprae* flowers, known for their potent antioxidant properties, metal-chelating abilities, and reducing effects, exhibit a significant dose-dependent inhibitory effect on both α-amylase and α-glucosidase. The authors suggest that these effects are related to the presence of flavonoids, such as quercetin and luteolin, which are recognized for their ability to bind to the active sites of these enzymes [46].

The antidiabetic effect of methanolic extracts from *O. pes-caprae* flowers was evaluated based on their ability to inhibit the non-enzymatic glycation process. The findings indicated that the extract had a significant inhibitory effect on forming early-phase glycation products, successfully reducing fructosamine levels in a dose-dependent manner [46].

Thiols, which play a role in various enzymatic reactions such as transcription, signal transduction, cell signaling, detoxification, and apoptosis, are susceptible to oxidation and glycation. The glycation of thiols can lead to the formation of protein cross-links, which may contribute to the complications associated with type 2 diabetes mellitus, such as vascular diseases [51]. Conversely, the oxidation of thiol groups in enzymatic antioxidants results in a loss of their activity, thereby increasing oxidative stress and exacerbating the progression of type 2 diabetes [52]. The inhibitory activity of *O. pes-caprae* polyphenols against AGE formation involves several mechanisms. These include scavenging free radicals, protecting protein thiol sites, inhibiting metal chelation, and reducing hyperglycemia by inhibiting the enzymes α-amylase and α-glucosidase [46,53,54].

The antidiabetic effect of the methanolic extract of *O. pes-caprae* was also confirmed in an alloxan-induced diabetes mouse model [46]. The oral administration of two doses of *O. pes-caprae* flowers per day for three weeks decreased malondialdehyde levels in the liver. Additionally, the treatment led to an increase in the activity of antioxidant enzymes such as SOD, CAT, GPx, and GR. Furthermore, the treatment enhanced the activity of enzymes involved in carbohydrate metabolism, including hexokinase, glucose-6-phosphate dehydrogenase, and pyruvate kinase, inducing a reduction in blood glucose levels, a restoration of normal metabolism through the stimulation of lipogenesis, and, consequently, an increase in body weight [46]. The substantial findings regarding the antihyperglycemic activity of *O. pes-caprae* indicate that this plant may be a promising candidate for the nutraceutical sector in terms of developing natural ingredients to improve or protect against diabetes and its complications.

### 6.3. Antibacterial and Antifungal Activities

The search for new antimicrobial agents is essential due to the growing issue of antibiotic resistance. Natural compounds are particularly promising because they have evolved to fight microbial competition in their natural habitats. As a result, these molecules often demonstrate strong effectiveness against resistant bacteria, including those classified as multidrug-resistant or extensively drug-resistant [55].

Ethanol, methanol, acetone, *n*-hexane, and chloroform extracts of *O. pes-caprae* at the flowering stage exhibited antibacterial activity against bacterial strains such as *Xanthomonas*, *Clavibacter michiganensis*, and *Bacillus* at concentrations of 1000, 1500, and 2500 ppm [29]. The ethanolic extract demonstrated the highest level of activity. At the same concentrations, the examined extracts also showed antifungal activity against fungal strains, including *Aspergillus flavus*, *Penicillium*, and *Fusarium solani*. The ethanolic extract at concentration 1000 ppm was particularly effective against *A. flavus*, followed by *F. solani* and *Penicillium* [29].

### 6.4. Vascular Activity

Consumption of polyphenol-rich foods is linked to a lower risk of hypertension and fewer cardiovascular events. It is reported that traditional medicine uses the leaves of *O. pes-caprae* for their antihypertensive effects and the roots for their diuretic activity [42,56]. The polyphenolic fraction of an *O. pes-caprae* L. leaf extract was tested for its vascular activity in the human internal mammary artery (HIMA). The extract, tested at a maximum concentration of 0.63 mg/mL, showed no inherent activity, as it did not exert any effect on the HIMA. However, the concentration of 0.66 mg/mL significantly reduced the maximum contraction response to noradrenaline by 58.44% (*p* < 0.05). These results point to a non-competitive antagonistic action at adrenergic receptors (α_1_ and α_2_), as evidenced by the absence of intrinsic activity and the diminished maximum contraction and potency of the agonist, and appear to be attributable to luteolin, apigenin, and their derivatives [43,57,58].

Subsequently, Fonesca and collaborators confirmed that the effects of the *O. pes-caprae* extract are not due to its affinity for the α_1_ and α_2_ adrenergic receptors but its affinity for the Beta-type receptors. A higher concentration of the extract (i.e., 10.4 mg/mL) is not able to inhibit the contractile response to noradrenaline [58]. 

### 6.5. Neuroprotective Activity

The potential neuroprotective effect of *O. pes-capre* extract against ischemic injury could be linked to the antioxidant capacity of the phytocomplex and, in particular, to some polyphenol derivatives such as homoorientin, orientin, and vitexin. These compounds are able to reduce the levels of lactate dehydrogenase induced by oxygen–glucose deprivation without causing the death of hippocampal neurons, as reported by Gaspar and collaborators [43].

## 7. Sustainable Agricultural Application

Studies have demonstrated that *O. pes-caprae* exhibits notable fertilizing properties. Incorporating fresh biomass (leaves and flowers) from *O. pes-caprae* into the soil following a decomposition period has significantly enhanced the growth and biomass of *Lactuca sativa* L. and *Diplotaxis tenuifolia* (L.) DC [59]. In particular, incorporating *O. pes-caprae* biomass into the soil has also induced a certain tolerance to water stress in *D. tenuifolia* [59]. Furthermore, the presence of *O. pes-caprae* has been found to influence soil phosphorus (P) levels, with invaded areas displaying higher concentrations of bioavailable P compared to non-invaded sites. This effect is attributed to the release of oxalic acid from the plant’s leaves during decomposition, where oxalate functions as a chelating agent that improves phosphorus solubility and accessibility in the soil [17].

## 8. Conclusions

In this study, we summarized the most relevant findings regarding the phytochemistry and biological activities of *O. pes-caprae*, an invasive species distributed worldwide. Invasive plants represent a critical ecological issue and an economic concern for agriculture. Moreover, the invasiveness of alien plants is often connected with climate change [60], showing that these environmental problems are strictly interconnected. IAS are usually undemanding plants that grow spontaneously and differently from other species, such as endangered ones, and it is possible to collect large amounts of them almost without concern, making them a significant source of potential bioactive compounds. Scientific research should explore and confirm the health benefits of invasive species and identify and optimize green extraction techniques to recover their bioactive compounds, thereby promoting the sustainable use of these plants.

Due to several logistical, financial, and ecological issues, IAS are used sparingly as raw materials in many different industries. The unpredictable nature of their supply is a significant worry. However, IAS are actively regulated and controlled to stop their spread and their supply is irregular and might not be sufficient to meet the needs of extensive industrial applications. While certain IAS have beneficial qualities, processing them can be expensive and complicated from a technical standpoint. Switching to IAS-based alternatives is less appealing since many sectors depend on well-established supply chains and standardized raw materials that guarantee quality, efficiency, and cost-effectiveness. From an ecological perspective, encouraging IAS in sectors may have unforeseen repercussions, such as broader proliferation, if their economic worth rises. However, using sustainable harvesting may have some advantages if it can be incorporated into the current control strategies. In the end, even though IAS may be utilized in some industries, these issues need to be thoroughly examined to make sure that their application is in line with both practicality and environmental responsibility.

The scientific literature data summarized in this review highlight *O. pes-caprae* as a promising source of phytochemicals that exhibit significant biological activities, including antioxidant, antimicrobial, and antidiabetic properties.

To our knowledge, few studies have investigated the phytochemical profile of *O. pes-caprae* and only one of them characterized its volatile compounds. The results reported above show that the quantitative and qualitative composition of *O. pes-caprae* extracts greatly varies depending on the time and geographic area of plant collection and the applied extraction technique.

Thus, considering the wide distribution of this invasive species, we encourage further studies on the phytochemical characterization and health-beneficial properties of this plant. Therefore, with this comprehensive review, we encourage deeper investigations of *O. pes-caprae* for a broader understanding of its potential as a medicinal and food plant, with the scope of turning this species from enemy to ally. We may decrease the ecological effects of invasive species and provide new economic opportunities by redefining them as advantages rather than problems. Strategic utilization of invasive alien species can transform environmental issues into conservation and economic development opportunities. 

## Figures and Tables

**Table 1:** Approximate composition of *O. pes-caprae* aerial parts [38].

Class (%)	Flowers	Leaves	Stems
Carbohydrates	62.6	45.9	73.3
Fats	6.7	12.7	3.5
Proteins	13.5	19.4	8.9
Dietary Fibers	30.7	28.7	36.4
